# Goth Music and Depressive Symptoms among Adolescents: A Longitudinal Study

**DOI:** 10.1007/s10964-020-01294-y

**Published:** 2020-08-19

**Authors:** Tom ter Bogt, William W. Hale, Natale Canale, Massimiliano Pastore, Alessio Vieno

**Affiliations:** 1 Department of Interdisciplinary Social Science, Utrecht University, Padualaan 14, 3584 CH Utrecht, The Netherlands; 2 Department of Pedagogics (Youth & Family), Utrecht University, Heidelberglaan 1, 3584 CS Utrecht, The Netherlands; 3 Department of Developmental Psychology and Socialisation, University of Padova, Via Venezia 8, 35131 Padova, Italy

**Keywords:** Adolescence, Depression, Emo, Goth, Gothic, Music preferences

## Abstract

Identification with Goth youth culture has been related to elevated levels of depression, self-harm and suicide ideation. However, this identification may be difficult to assess as Goth is stigmatized and adolescents may hesitate to self-identify. Conversely, adolescents readily respond to questions on their music preferences. This study addresses the potential link between liking Goth music and depressive symptoms in a four-year study among 10 to 15-year-olds (*N* = 940, *M* age = 12.4 at T1, 49% female). In this study, it was found that Goth music is only liked by a small minority of adolescents (4 to 11%). Both girls and boys who liked Goth music reported increased levels of depressive symptoms as they grew older. The findings of this study suggest that a preference for Goth music emerges as an early, sensitive marker of dormant or developing depressive symptoms in adolescents. The mechanisms through which music preferences can translate into or sustain depressive symptoms are discussed.

## Introduction

Depression among adolescents is a commonly occurring disorder that causes much impairment to youth (Kessler et al. 2012). It has been noted that the relatively low prevalence level of depression in children spikes to high levels in adolescence, especially for females (Zahn-Waxler et al. 2008). In order to better understand this sudden rise in depression occurrence in adolescents, the influence of several psychosocial factors has been proposed. One such influence is that of the adolescent’s peer group (Parker et al. 2006). As a part of peer group belongingness, music preferences play a role in determining group inclusion, in addition to other aspects such as appearance and behaviors (Van Zalk et al. 2011). It is for this reason that music preferences, as a psychosocial factor, have been related to the development of adolescent depressive symptoms. This study explores why and how early adolescent music choices, in particular for Goth music, may function as gender specific markers of subsequent rises in adolescent depression symptomology.

### Origins and Characteristics of Goth Music

Goth music and youth subculture originate in the British punk and new wave scene of the late seventies and eighties. Both girl and boy fans of bands such as the Cure and Siouxie and the Banshees dressed all-black, dyed their hair black, and put on black make up, to emulate the fashion choices of the band members. They often shared a gloomy outlook of the world, in general, and their personal lives, in particular, reflecting the esthetics and tone of the appearance and lyrics of their favorite bands (Hodkinson 2002). In the USA, both Goth music and its subculture gained notoriety with the ascent of Marilyn Manson (a conjunction of the names of movie star Marilyn Monroe and mass murderer Charles Manson). With a loud, harsh, bleak mix of music and lyrics Marilyn Manson became one of the most successful bands of the late nineties and early new century, even though the esthetics, attitudes and behaviors of its protagonist “Marilyn Manson” (Brian Hugh Warner) were highly provocative (Wright 2000). Albums with titles such as Antichrist Superstar (1996), Mechanical Animals (1998), and Eat Me, Drink Me (2007) were bestsellers in the US and popular across Europe, Australia and in Japan. Not all bands in the Goth scene can be characterized by the melancholic, nihilistic outlook as exhibited in Marilyn Manson’s appearances and utterances, but it is fair to conclude that Goth music and subculture emanate a romantic-pessimistic outlook of not-being-in-place-in-this-world, and longing for a different one (Rutledge et al. 2008). Billie Eilish, drawing from Goth’s style catalog, covering themes ranging from climate change –“hills in California burn”– to suicide of peers—“friends I had to bury”—has become a neo-Goth superstar, appealing to young people alienated from mainstream American, that is, western consumer culture and its proponents (Haring 2019).

### Goth and Problem Behavior

Goth subculture has become a haven for adolescents who are proportionally more at odds with themselves, their parents and teachers, other authorities and the world at large than most of their peers (Rutledge et al. 2008; Young et al. 2006). It must be noted that for adolescents there is comfort in meeting similar spirited people who provide support in coping with personal problems and dealing with a social environment that may be perceived as non-inviting or even hostile (Bešić and Kerr 2009; Miranda 2013). Nevertheless, in a small body of research it has been established that a substantial minority of Goths exhibit more problems than their non-Goth peers. Identifying with a “radical crowd”, such as Goth, implies creating a distinctive appearance that may come across as startling or even shocking to non-members. It is paradoxical that particularly shy young people pursue such a radical appearance, but it may help them in at least two ways. Adopting a Goth style may limit their social contacts to peers with the same style and signal to more mainstream oriented peers that they do not aspire contact. Goth style, thus, creates a small, familiar group of likeminded peers. Rejection from peers outside this group can then be attributed to the dislike of their highly identifiable looks, setting them apart from these peers, and not blamed on their shyness (Bešić and Kerr 2009). However, adopting a Goth appearance does not seem to be productive for enhancing self-esteem and buffering depression. Adolescent “shy radicals” were not more inhibited than shy adolescents in other crowds, but that they were more depressed and showed lower self-esteem than inhibited youths in other groups

In a longitudinal cohort study among adolescent Goths, the authors did not address depression per se, but related factors such as self-harm and suicide attempts (Young et al. 2006). Scottish young people identifying with Goth youth culture were more inclined to self-harm and attempted suicide than those not identifying with this subculture. Analyses in which Goth identification was compared to affinity with fourteen other subcultures, showed that Goth emerged as the primary subcultural identification linked to the lifetime prevalence of these problems. Adjusting for confounders such as gender, having divorced or separated parents, smoking, drug use, and prior depression did not attenuate Goth’s effects. Goth identification remained the most important factor indicating self-harm and suicide attempts. The immersion in Goth culture may instigate or worsen problems, but young people with problems may also choose Goth as a scene sensitive to and reflective of problems they have already encountered. The relation between Goth identification and problem behavior link may, thus, be the result of processes of both influence and selection (Young et al. 2006).

In a longitudinal, comprehensive study on Goth, depression and self-harm in the UK, it was found that among 15-year-olds, 18 and 37% of the adolescents self-identifying as Goths reported depressive symptoms or self-harm, respectively, compared to 6 and 10% of their non-Goth peers (Bowes et al. 2015). Identifying with Goth emerged as a powerful predictor for depression and self-harm at age 18. Compared with non-Goths, those who identified with Goth were 1.6 to 3.7 times more likely (unadjusted ORs) to meet the criteria for depression. The corresponding ORs for self-harm were 1.5 to 3.7. Risk factors such as emotional and behavioral problems, peer victimization and maltreatment may propel adolescents in the direction of subcultures addressing and reflecting their experiences, worries and problems. But in models controlling for these factors, adolescents identifying with Goth at age 15 still showed increased risk of depression and self-harm at age 18: Goths represent a vulnerable group (Bowes et al. 2015).

### Music Preferences as a Marker of Later Problem Behavior

Studies such as quoted above have suggested that a selection effect is at work: adolescents with problems are attracted to non-mainstream sub-cultures and music scenes that reflect these problems. Other scholars have proposed peer mediation as a mechanism explaining the relation between liking certain types of music and increased problem behavior, such as substance use and abuse. Both cross-sectional (Mulder et al. 2010) and longitudinal studies (Slater and Henry 2013) tested the hypothesis that music preferences drive involvement with substance using peers, resulting in less of more substance use, according to the character of these peer groups. Thus, both selection on the basis of music preferences and peer influence may explain the music-problem behavior link.

Music Marker Theory (Ter Bogt et al. 2013) addresses music’s selection effects and peer influence processes, and additionally, potential direct media influences. This theory starts with the notion that music preferences are already in place early in adolescence and remain highly stable across adolescence (Delsing et al. 2008). It is furthermore important to note that adolescent externalizing problem behavior peaks in mid to late adolescence, and that internalizing behaviors increase across adolescence, particularly among girls, implying that music preferences precede much problem behavior (Zahn-Waxler et al. 2008). Music Marker Theory poses that through music preferences adolescents seek friends and become members of crowds that have a more or less deviant reputation (social selection) (Selfhout et al. 2008, 2009). Within these friendships and crowds, adolescents may be affected by either pro-social or problematic models, a process that has been described as contagion (Dishion and Tipsord 2011). With regard to depressive symptoms concepts have been introduced such as socialization of depressive symptoms (Conway et al. 2011), or convergence to group standards in depressive behavior (Kiuru et al. 2012) (social transmission). Whereas adolescents with mainstream music tastes, in mainstream crowds, may develop little problem behavior, the opposite may be more characteristic of those with non-mainstream music tastes and attracted to non-mainstream crowds (Mulder et al. 2010; Slater and Henry 2013; Tanner et al. 2008; Ter Bogt et al. 2013). In addition to these selection and transmission effects Music Marker Theory does not rule out direct media influences. Music lyrics can function as messages to adopt attitudes or imitate behaviors and listening to sad music may result in sustained rumination, and as a consequence depressive mood (Garrido and Schubert 2015).

Music Marker Theory’s proposed chain of event already has been well documented in regard to externalizing problem behaviors. An early and lasting preference for non-mainstream music such as heavy metal and punk, or extreme forms of hip hop and dance, such as gansta-rap and hardhouse, foreshadow more frequent externalizing problem behaviors later in adolescence. In other words, early music preferences can function as *markers* for concurrent and later externalizing problem behaviors (Franken et al. 2017; Ter Bogt et al. 2013). This theory has received much less study in regard to internalizing problem behaviors.

### Studying Music and Gender

Previous studies of Goth preference in adolescence have relied on measures of adolescents’ self-measurements of their cultural orientations. But these orientations may be difficult to assess and theoretical conceptualizations such as Music Marker Theory indicate that a shift to investigating music preferences in relation to adolescent problem behavior may be worthwhile and open up a new avenue for research. Measuring youth cultural orientations is challenging. Some adolescents may be proud to identify with crowds with a certain reputation and style, while others may be reluctant to do so and describe themselves as being either “normal”, “themselves”, or “an individual” and not identify themselves with a particular group (Ter Bogt et al. 2013). Conversely, adolescents may think or aspire to belong to a group with a certain reputation, but not belong to it in the eyes of others. Self-identifying or, conversely, not-identifying with a particular group does not necessarily indicate one’s reputation among peers (Cross and Fletcher 2009). With regard to a highly visible and non-mainstream group such as Goth, a hesitancy of the adolescent to identify with it may be strong as adolescents who do identify themselves as Goths feel stigmatized (Bešić and Kerr 2009). Hence, these studies may systematically underestimate the number of young people that identify either openly or privately with Goth.

Assessing music preference is easy. Adolescents readily respond to questions on the music that they like or dislike. Obviously, preferring music is not the exact same as identifying oneself as member of the Goth crowd. However, by using music preference measures a potentially broader group that identify with Goth culture and Goth music can be researched. Additionally, it should be noted that music preferences take on a more defined form in early adolescence and are relatively stable across adolescence (Delsing et al. 2008). Investigating music in relation to problem behaviors is not only relevant theoretically, it also provides the opportunity to focus on a young group of adolescents and follow them through adolescence to understand how the relation between their music preferences and problem behaviors unfolds.

In earlier studies no specific attention was given to gender differences. Many studies have shown that across adolescence internalizing problem behaviors increase much more in girls compared to boys (Hilt and Nolen-Hoeksema 2013). And since the late 1980’s it has been noted that gender is central to the ways in which popular music tastes are formed (Christenson and Peterson 1988). Therefore, in the study of music in relation to depressive symptoms, gender is a crucial factor to be accounted for.

## Current Study

This study deviates from earlier studies on Goth and internalizing problem behaviors, such as depression, in four respects. First, earlier studies employed measures of *youth cultural orientation*, whereas in this study *music preference* is modeled as the most important predictor of depressive symptoms. Music is at the heart of Goth culture and measuring adolescents’ likes and dislikes of this music may be a less intrusive way to identify adolescent Goths. Second, modeling music taste, which is already in place in early adolescence, allows for the study of a younger sample than in previous studies and follow them through adolescence. Third, in previous studies no specific attention was given to gender differences. Gender differences in depression emerge in adolescence and gender is also a key factor in music preferences and their structure. It is therefore important to study gender’s potential moderating role in the association between music and internalizing problem behaviors, such as depression. Finally, earlier longitudinal research on the Goth and depression link did not account for the fact that observations are nested within individuals and may therefore have led to misspecification of results. In the current multi-level approach, growth in music preferences is tracked in relation to depressive symptoms in order to answer the following research question: Does preferring Goth music predict depressive symptoms across adolescence, and does gender moderate this relation?

## Methods

### Study Design and Participants

Participants in this study were 940 adolescents of the early adolescent cohort (10 to 15-year olds) participating in the CONflict And Management Of RElationships study (CONAMORE) (Meeus et al. 2004). CONAMORE is an ongoing longitudinal study that examines the relationships of Dutch adolescents with parents and peers as well as their emotional states. In the current study, data were used from four waves with a 2-year interval between wave 1 and 2, and 1-year intervals between wave 2, 3 and 4. Of the 940 participating adolescents 51% were male and 49% were female, mean age at T1: 12·4 years. Sample attrition was 1.2% across the waves.

### Procedure

Participants came from various high schools in Utrecht and surroundings. Parents and students received a letter in which the aims of the study were described, and information was given about the option of not participating. Students were required to provide written informed consent. Less than 1% decided not to participate. Participants completed a series of questionnaires in their classroom after school hours. Research assistants, who attended the administration, gave verbal instructions about filling out the questionnaires; written instructions were also included. Confidentiality of their given answers was guaranteed explicitly. For students who were absent on the day of testing, a second assessment time was organized. Students who were absent on both days of testing were not assessed. At each wave, respondents received 10 euros, after completing the questionnaires.

### Measures

#### Background variables

Respondents reported on their gender, age and education level (vocational training vs. pre-academic education).

#### Goth music preferences and self-identification

Adolescents’ music preferences were assessed by means of the Music Preference Questionnaire (MPQ) (Sikkema 1999). The MPQ consists of a list of 13 established categories of music, Goth music one of them. Subjects were asked to indicate on five-point Likert scales “the extent to which they liked” each of the genres listed (from 1 = *do not like at all* to 5 = *like very much*). Option 6 indicated *do not know this type of music*. These scores were treated as missing values that were estimated in the analyses. In order to assess whether Goth music preference is linked to Goth youth culture respondents were furthermore asked to indicate the extent to which they identified with Goth subculture and believed it suited them. Five-point Likert scale ranging from 1 = *does not suit me at all* to 5 = *suits me very well*.

#### Depressive symptoms

The Children’s Depression Inventory (CDI) is a widely used self-report questionnaire of depressive symptomology in children and adolescents aged 8 to 18 years (Timbremont and Braet 2002). The questionnaire is composed of 27 items that review the various depression symptoms categories such as mood, vegetative, cognitive, and psychomotor disturbances. The questionnaire is scored on a three-point scale ranging from 1 *not true*, to 2 *a bit true* and 3 *very true*. The total scores on the questionnaire can range from 27 to 81, with higher scores being reflective of greater depression. The CDI has strong internal consistency and validity in nonclinical populations (Saylor et al. 1984).

The factorial structure of depression in each wave was evaluated through Confirmatory Factor Analysis (CFA), applying the R package lavaan (Rosseel 2012). As item scores consist of three discrete/ordered values, the Robust Diagonally Weighted Least Squares estimator was used. In Table 1 the fit indices of models from the four subsequent waves are presented: Comparative Fit Index (CFI; (Bentler and Bonett 1980), Nonnormed Fit Index (NNFI) (Tucker and Lewis 1973); Root-Mean-Square Error of Approximation (RMSEA; Steiger and Lind 1980), and the Total Coefficient of Determination (TCD) (Bollen 1989). Across all four waves the models show satisfactory fit values. Subsequently, factor scores of depressive symptoms were calculated for each wave. These factor scores (modeled as observed variables) were used in the follow-up analyses (see section below).

**Table 1:** Fit indices of confirmatory factor analysis models of depressive symptoms

Variable	Wave	*N*	CFI	NNFI	RMSEA	TCD
Depressive symptoms	1	848	0.990	0.989	0.047	0.984
Depressive symptoms	2	901	0.979	0.978	0.053	0.977
Depressive symptoms	3	901	0.981	0.980	0.054	0.981
Depressive symptoms	4	889	0.978	0.976	0.052	0.976

Values for good/excellent fit and model quality: CFI, Comparative Fit Index >0.97; RMSEA, Root Mean Square Error of Approximation <0.05; NNFI, Non-Normed Fit Index (>0.95; TCD, Total Coefficient of Determination estimates the amount of explained model variance and ranges from 0 (i.e., 0% of variance explained) to 1 (i.e., 100%)

### Strategy for Analysis

#### Generalized Linear Mixed Models

To explore the effects of predictors on depressive symptoms, a set of Generalized Linear Mixed Models was conceptualized (Hedeker 2005; Faraway 2006). These models allow for: (1) modeling the dependent variable with accuracy, given that it is skewed; (2) obtaining adjusted estimates for repeated sampling (i.e., when a series of observations is nested within an individual); (3) countering sampling imbalance; and (4) countering variation among individuals within the data (McElreath 2015). To take all the advantages of the modeling procedure, parameters were estimated by adopting the Bayesian approach, implying a focus on the (posterior) probability of a set of parameters, given the observed data and priors and estimated the relative evidence of different models using Information Criteria. To model the longitudinal links between preferring Goth music and depressive symptoms for both genders, several mixed effects models were tested, including all combinations of predictors (gender, Goth, years), with subjects as random effects and random slopes of years within subject. More specifically, starting from the null model (no predictors) and all the predictors and their interactions were subsequently introduced. In preliminary analyses education level was introduced as a control variable, but as this factor did in no way affect the results, it was left it out of the definite analyses.

#### Model fitting and parameter estimation

Models were fitted by using the Bayesian MCMC estimation method implemented in the probabilistic programming language STAN through the R package brms (Buerkner 2016). For the regression parameters (ß) normal priors (mean = 0 and sd = 10) were introduced, and for standard deviation parameters the half-Student t distribution (df = 3, mean = 0, sd = 10) was used. Posterior distributions for each parameter were estimated using four MCMC chains each replicated 2000 times. MCMC convergence was assessed by calculating the potential scale reduction statistic (PSRF) (Gelman and Rubin 1992); this statistic measures the ratio of the average variance of samples within each chain to the variance of the pooled samples across chains; chains are considered at equilibrium with values around one.

To compare models the Widely Applicable Information Criterion was examined (WAIC, lower values indicate a better fit) (Watanabe 2010); and, in addition, the Akaike Weight, i.e., an estimate of the probability that the model will make the best prediction on new data, conditional on the set of models considered (McElreath 2015). To profile the best model data were summarized by providing posterior means and 95% Credibility Intervals (CI) (Kruschke 2011).

## Results

### Descriptive Statistics and Correlations

Table 2 shows the descriptive statistics of the sample with regard to their knowledge of and preferences for Goth music, and depression. It is noticeable that Goth music is a relatively unknown genre at T1 (44.4%), but that familiarity increases rapidly, with only 2.4% not knowing this type of music at T4. Goth music is liked by a small minority of young people. Among girls, the range of those preferring it much or very much ranges from 4.3% at T1 to 7.5% at T4, among boys from 10·8% (T1) to 3·8% (T4). Goth music is particularly popular at age 14–15, that is, when Goth has become a better-known genre (10.8% of respondents favoring Goth (very) much). At age 16–17 only a small minority of adolescents still likes this type of music (much) (5.5%). Girls reported a higher preference for Gothic music than boys in wave 2. Across wave 2 to 4, girls show more depression than boys.

**Table 2:** Music preferences and depressive symptoms by gender

Variable	Wave	Gender		All	Knowledge
		Girl	Boy	Total	% Do not know
% Liking or Very much liking Goth music	1	4.3	10.8	7.5	44.4
	2	13.2^a^	8.4^b^	10.8	6.0
	3	9.3	6.5	7.9	4.2
	4	7.5	3.8	5.5	2.4
Mean score and SD preference for Goth music (range 1–5)	1	2.00 (1.08)	2.20 (1.22)	2.11 (1.16)	
	2	1.95 (1.22)^a^	1.73 (1.03)^b^	1.84 (1.13)	
	3	1.76 (1.12)	1.64 (0.99)	1.70 (1.05)	
	4	1.69 (1.04)	1.53 (0.85)	1.61 (0.94)	
Mean score and SD Depression (range 1–3)	1	1.17 (0.21)	1.16 (0.30)	1.16 (0.26)	
	2	1.21 (0.25)^a^	1.15 (0.23)^b^	1.18 (0.24)	
	3	1.22 (0.26)^a^	1.15 (0.21)^b^	1.19 (0.24)	
	4	1.21 (0.24)^a^	1.14 (0.20)^b^	1.18 (0.22)	
*N*		461	476	937	

Dissimilar row superscripts indicate differences between girls and boys with regard to liking Goth music or depressive symptoms (*t*-test)

*p* < 0.01

It is furthermore interesting to note that the answers to questions on Goth music preference were compared with reports of Goth youth culture identification across four waves. Results revealed that correlations between Goth music preference and Goth youth cultural identification range from 0.51 to 0.72 (not in Table). But, whereas 5.5 to 10.8% of the respondents reported to “like” or “very much” like Goth music, only 2.8–5.3% indicated that Goth youth culture “suits me” or “suits me well”, substantiating the assumption that music and youth culture are strongly linked, but that Goth music is more popular and regards a larger group of adolescents than Goth youth cultural style. Table 3 shows the Pearson correlations between Goth music preference and depressive feelings across four waves. Results show that particularly at T2, T3 and T4 significant correlations exist between liking Goth and depression, and that these relations do not differ much for girls (range 0.17–0.26) compared to boys (range 0.12–0.22).

**Table 3:** Pearson correlations between Goth music preferences and depressive symptoms at four waves

	1	2	3	4	5	6	7	8
1. T1 Goth		0.34*	0.35*	0.24*	−0.02	−0.04	0.04	0.12
2. T2 Goth	0.07		0.64*	0.60*	0.23*	0.17*	0.28*	0.17*
3. T3 Goth	0.15*	0.51*		0.72*	0.11	0.17*	0.26*	0.25*
4. T4 Goth	0.09	0.35*	0.56*		0.18*	0.20*	0.30*	0.25*
5. T1 Depression	0.12	0.19*	0.12	0.06		0.47*	0.38*	0.36*
6. T2 Depression	−0.05	0.14*	0.09	0.06	0.25*		0.58*	0.61*
7. T3 Depression	0.04	0.14*	0.20*	0.13*	0.19*	0.35*		0.62*
8. T4 Depression	0.02	0.03	0.14*	0.22*	0.20*	0.29*	0.49*	

Above the diagonal values for girls; underneath values for boys

*p < 0.01

### Generalized Linear Mixed Models

**Fig. 1:**
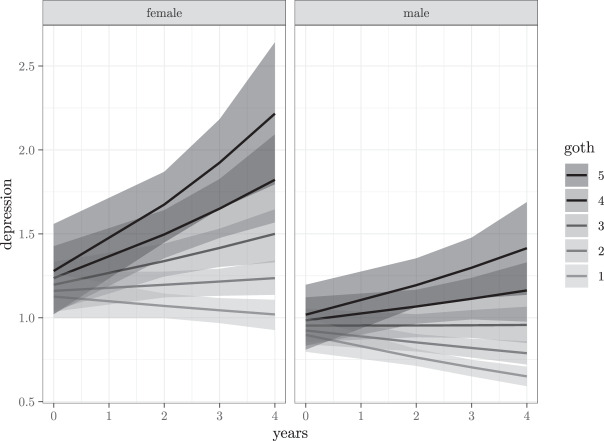
Depressive Symptoms as a function of Liking Goth Music (different lines) and Years, Separately for Gender (different panels). Goth: 1 = “Do not like at all” to 5 “Do like very much”. The lines represent expected values of depressive symptoms on the basis of factor scores in the empirical data ranging from 0.008 to 4.89. The shaded regionsrepresent the 89% Highest Posterior Density interval

Table 4 presents the fit indices of the eight tested models. At the start the null model (M0), the model with no predictors, was fitted and then all other predictors were added, one by one. M1 to M5 regard random intercepts models. First Goth preference was included (M1), subsequently, Goth and years (time)(M2), the interaction between Goth * years (M3), this last interaction plus gender (M4), and the Goth * year and gender * years interactions (M5). M6 and M7 regard random intercepts and slopes models with the Goth * year and gender * years interactions (M6), and finally the three-way interaction between Goth * gender * years (M7). As can be noticed in Table 4 the best model was the one (M6) with two interactions, Goth * years and gender * years, and added intercepts and slopes varying within subjects. This model is characterized by a weight of 97%, indicating the high probability of the model to predict new data in comparison to the other models, and conditioned on the basis of the observed data. This weight is much higher than that of the other models considered (Burnham et al. 2011). More in detail, model M6, weight = 0.97, is about 32 times more plausible than model M7, weight = 0.03) and much more than the other models. In Table 5 the estimated parameters of model M6 are reported. The results demonstrate that females show more internalizing problems that males (PM gender = 0.23, SD = 0.09). Within the context of this study it is most notable that across adolescence Goth fans develop more depression than their non-Goth peers (PM Goth * years = 0.04, SD = 0.01). Figure 1 shows that those who (strongly) like Goth music, as indicated by the blue and violet lines, are at an increased risk for depressive feelings. It is furthermore relevant to note that adding a three-way interaction, Goth * gender * years (Model 7), in addition to random intercepts and slopes, did not, as noted before, increase model weight. In other words: the lines in Fig. 1 may seem to indicate differences in the gradient of the goth music-depressive feelings link between girls and boys, but these differences are weak. Both female and male Goth fans are more inclined to depressive feelings across adolescence, compared to adolescents not liking this type of music.

**Table 4:** Model comparison table

	Models	WAIC	SE	^δ^WAIC	Weight
M6	Dep ~ Goth * years + gender * years + (years | subj)	6036.64	102.95	0	0.97
M7	Dep ~ Goth * gender * years + (years | subj)	6043.87	102.80	7.23	0.03
M5	Dep ~ Goth * years + gender * years + (1 | subj)	6065.80	101.36	29.16	0.00
M4	Dep ~ Goth * years + gender + (1 | subj)	6075.00	100.91	38.36	0.00
M3	Dep ~ Goth * years + (1 | subj)	6079.88	100.30	43.24	0.00
M1	Dep ~ Goth + (1 | subj)	6084.36	99.95	47.72	0.00
M2	Dep ~ Goth + years + (1 | subj)	6085.07	100.08	48.43	0.00
M0	Dep ~ (1 | subj)	6086.23	100.30	49.59	0.00

*Dep* Depressive symptoms, *WAIC* Widely Applicable Information Criterion; ^δ^WAIC = difference between each WAIC and the lowest WAIC; Weight = Akaike Weight; (1 | subj) = random subject intercept; (years | subj) = random slope of years within subject

**Table 5:** Parameters for Model M6: Goth music and depressive symptoms across years

	PM	SE	95% CI	PSRF
Fixed effects				
Intercept	−0.14	0.10	−0.34/0.04	1
Goth music	0.03	0.04	−0.04/0.10	1
Years	−0.12	0.03	−0.18/−0.05	1
Gender	0.23	0.09	0.06/0.40	1
Goth music * Years	0.04	0.01	0.01/0.07	1
Gender * Years	0.06	0.03	0.00/0.11	1
Random effects				
Sd (intercepts)	0.41	0.06	0.29/0.51	1
Sd (years)	0.05	0.02	0.00/0.09	1
Cor (intercept, years)	0.69	0.32	−0.23/0.99	1

*PM* posterior mean; *SE* standard error of the posterior mean; *CIs* credibility intervals; *PSRF* potential scale reduction factor, at convergence: PSRF = 1

## Discussion

During the last two decades Goth has developed into a highly visible youth cultural scene for young people attracted to black-romantic music and esthetics. Earlier studies have shown that identifying with of being part of the Goth scene (in the UK) may indicate serious internalizing problem behaviors such as depression, self-harm and suicide attempts. This study set out to explore the potential association between, specifically, liking Goth music and depressive symptoms, as adolescent music preference is easier to measure (both for adolescents and researchers alike) than the more abstract concept of youth culture. An advantage of assessing Goth music preference as opposed to Goth youth culture may be that a group is captured that may not be “hardcore” Goth stylistically, but attracted to the themes present in Goth music, its lyrics, and visualization. Furthermore, the understudied potential moderating role of gender in the link between Goth and depressive symptoms was analyzed. Results show that, indeed, the adolescent group reporting to like Goth *music* is larger than the one indicating that Goth *youth culture* suits them. The most important finding is that Goth music preference indicated depressive symptoms across adolescence, but that gender did not moderate this relation.

Analysis of the data of a four-wave longitudinal study brought forward evidence that at the onset of adolescence, mean age of the respondents 12.4 years, Goth was not a well-known music genre. But during the two years thereafter, the obscurity of Goth music vanished at a fast pace. At the time of the second wave of the study, the respondents were on average 14.4 years of age and Goth was not only a well-known genre, it was also relatively popular. In mid-adolescence the popularity of Goth music peaked with about 1 in 10 adolescents reporting (somewhat) liking this music. Two years later, at T4, mean age 16.6 years, only about 1 in 20 (still) liked Goth.

More important in the context of this study is the finding that across adolescence Goth preference becomes associated with depression. In the correlational study no relation between liking Goth and depressive symptoms was found at T1, but this association became more salient across adolescence, i.e. in later waves of the study. In addition, results of the GLMM analysis showed a clear Goth * years interaction indicating that across time youths into Goth music were more inclined to depressive tendencies than their peers who did not particularly value or even disliked this type of music. These results, thus, corroborate earlier research showing that a subcultural identification with Goth—implying liking the music that is a vital part of this subculture—is associated with serious internalizing distress. This association is present in the UK (Bowes et al. 2015; Young et al. 2006) and now shown to be also present in the cultural context of the current study, The Netherlands. Goth music may have an equal appeal cross-culturally and by attracting the same type of cultural “radicals”, relates to similar internalizing problems regardless of cultural context (Bešić and Kerr 2009). In addition, the results show that depressive tendencies are more prevalent in girls, a finding that has been brought forward in a large number of other studies (Hilt and Nolen-Hoeksema 2013), but no salient Goth * gender * years interaction was discerned. In other words, liking Goth does not increase depressive symptoms more in girls when compared to boys.

Earlier research suggests that depressive adolescents may be drawn disproportionally to Goth youth subculture. A selection effect may therefore explain the relation between Goth and depression (Bowes et al. 2015). This interpretation is in line with the finding that in early adolescence the link between liking Goth music and depressive symptoms is not present but develops across adolescence. Indeed, troubled young people may be fascinated by the dark symbolism and gloominess of Goth music and find relief in the fact that, for example, one of its most expressive proponents, Marilyn Manson, is not shy to expose his self-inflicted wounds and wade in misery in his lyrics, artwork and his biography *The Long Hard Road out of Hell* (Manson and Strauss 1998). Goth music may be comforting as it addresses difficulties that they may face themselves. They may be attracted by the colorful clothes and hairdos of Goths and even their outsider status, signaled by their appearance, may be appealing, as it reflects their own feelings of not fitting in the regular crowd system.

It must be noted though that this study started from another vantage point. Already early in adolescence most young people know different types of music well. They further have developed more or less outspoken preferences for specific music types. Due to their choice of music, adolescents may be attracted to and befriend other young people who like the same music. Thus, a selection effect is at work, but now on the basis of music preference, and not on the basis of depression. Goth music preference may lead to Goth crowd membership. Once part of the Goth crowd, an altogether different mechanism may occur. Goths may become more similar; the Goth crowd is a basis for socialization and members may model or dictate the looks, ideas and feelings of their peers. Thus, not only selection but also socialization is relevant for social anxiety in Goth and punk subcultures. It has been noted furthermore that adolescents in these crowds became isolated from the rest of the social network over time, as they not only befriended others outside their crowd to a lesser degree but were also being targeted less as a friend (Van Zalk et al. 2011). Our results also hint at the possibility that a similar mechanism is at work for depressive symptoms. Across adolescence, the popularity of Goth music decreases, but among its fans depressive symptoms increase. This substantiates the assumption that Goth music fans become more “hardcore” as time passes, with remaining fans ever more similar, not only in looks and values, but also in subculturally socialized and legitimized depressive feelings.

These results corroborate a crucial element of the Music Marker Theory. A preference for Goth indeed predicts later differences in adolescent internalizing distress. In that sense the results further validate a more general rule: stronger and more persistent identifications with non-mainstream music and non-conventional crowds are associated with more problem behaviors throughout adolescence (Prinstein and La Greca 2002; Doornwaard et al. 2012). In contrast, stronger and more persistent identifications with mainstream music and conventional crowds are generally associated with fewer problems (Ter Bogt et al. 2013). In future studies it would be worthwhile to track other non-mainstream types of music and their function as an early marker for internalizing problem behavior. Now that Goth seems past its heyday in the late nineties and early new century, other non-mainstream crowds should be the object of study. Emo, another music type and subcultural scene within the rock spectrum, dating back to American punk and hardcore of the eighties and nineties, is a likely candidate (Kiuru et al. 2012; Baker et al. 2013). Less black in appearance with fans wearing neon dyed hair and fantasy-movie inspired outfits, Emo subculture and its key elements Emo music and lyrics is characterized by a similar romantic out-of-place-ness as Goth and, potentially, corresponding health risks. More recently Billie Eilish has become a superstar with looks and attitudes referring back to Goth subculture, addressing teenage angst, loneliness and depression in her lyrics (Haring 2019).

Clinicians and professionals working with youth should, thus, be aware that non-mainstream preferences may indicate problems. This does not only apply to non-mainstream music in the rock, hip-hop or dance music traditions. For example, in a correlation study on music and internalizing distress a link between on the one hand liking complex non-mainstream music (classical music, jazz) or liking a broad range of types of music (omnivorous music preference) and, on the other hand, withdrawn behavior and anxiousness was found (Mulder et al. 2007).

This study regarded a large number of young people whose development was assessed longitudinally. Still, it did not directly address socialization of depressive symptoms (Conway et al. 2011) or convergence to group standards in depressive behavior (Kiuru et al. 2012). Finding the exact mechanics behind an adolescent’s choice of a subcultural group on the basis of his/her music preferences and the influence that the social norms and culture of that group on the adolescent is a valid topic for future studies. Additionally, it was not addressed whether the Goth-depressive symptoms link was due to Goth music socializing adolescents into depressive symptoms or depressed adolescents selecting Goth music (Young et al. 2006). However, this study’s modeling strategy was based on findings from earlier studies that found music predicting later adolescent problem behavior and not vice versa (Franken et al. 2017; Selfhout et al. 2008; Slater and Henry 2013; Ter Bogt et al. 2013). Therefore, Goth music was introduced as a predictor in the models. Third, no extended set of confounding variables was introduced. Obviously, gender was included as this study focused on differences between girls and boys in the link between music preferences and depression. Furthermore, educational level was tested, but including this factor in the models did not change or attenuate the results. It has to be noted though, that other studies included a strong set of confounders such as social class, substance use, maternal depression, exposure to life events and childhood peer victimization, but found that these factors did not (strongly) attenuate the link between belonging to the Goth scene and internalizing distress (Bowes et al. 2015; Young et al. 2006). In addition, early depressive symptoms have been shown to be a solid predictor of later depressive symptoms in adolescence (Garber et al. 2002). In the current study, earlier depressive episodes were accounted for, hence, it teased out Goth music preferences’ unique contribution in predicting depressive feelings later in adolescence. Fourth, a single item measure for Goth music preference was used instead of a scale. However, the music preference questions were straight forward and well understood by respondents. Repeating them in a slightly different format, with thirteen preferences to be measured with the MPQ, may irritate the adolescent respondents who might find these additional questions to be redundant. The MPQ items have low numbers of missing values even though the MPQ questions were located at the end of the survey. Cross-wave consistency in answering patterns on music items also indicates strong construct validity and test-retest reliability. Fifth, a statistical framework was utilized that is sensitive to the longitudinal data being nested within persons and the data were analyzed with state-of-the-art Bayesian methods. The final model showing that Goth is indeed linked to depression across time is plausible, still, the coefficients do not depart from zero dramatically, suggesting weak effect sizes. It must be noted though that these effect sizes are rather common in psychological research (Stanley et al. 2018). It must furthermore be noted that these weak associations may be accounted for by the fact that most Goth fans listen to Goth as they simply like the music, and that among these music fans internalizing problems may not be present. These weak, but significant associations hint at a subgroup within the Goth music fanbase showing more depressive symptoms than their Goth or non-Goth peers.

## Conclusion

While earlier studies have found a positive longitudinal relation between Goth *subculture self-identification* and depression, self-harm and suicide attempts, this study is the first to research Goth *music* preference in relation to depressive mood. A preference for Goth music emerges as easy obtainable, unobtrusive, and sensitive marker of dormant or developing depressive mood. When working with youth preferring Goth or other types of non-mainstream music, clinicians and other practitioners should be sensitive to the fact that music may mask problem behaviors. Music and its lyrics generally positively enhance an adolescent’s mood, help to develop an identity and provide comfort, but it is relevant to find out which music scenes do not buffer but enhance internalizing problem behaviors, and find out the exact mechanisms through which a beneficial medium can have detrimental effects.
